# Contraceptive Use in Premenopausal Women With Early Breast Cancer

**DOI:** 10.1001/jamanetworkopen.2022.33137

**Published:** 2022-09-23

**Authors:** Matteo Lambertini, Claudia Massarotti, Julie Havas, Barbara Pistilli, Anne-Laure Martin, Alexandra Jacquet, Charles Coutant, Florence Coussy, Asma Dhaini Mérimèche, Florence Lerebours, Christine Rousset-Jablonski, Christelle Jouannaud, Olivier Rigal, Marion Fournier, Patrick Soulie, Maria Alice Franzoi, Lucia Del Mastro, Ann H. Partridge, Fabrice André, Ines Vaz-Luis, Antonio Di Meglio

**Affiliations:** 1 Department of Medical Oncology, U.O. Clinica di Oncologia Medica, IRCCS Ospedale Policlinico San Martino, Genova, Italy; 2 Department of Internal Medicine and Medical Specialties (DiMI), School of Medicine, University of Genova, Genova, Italy; 3 Department of Neurosciences, Rehabilitation, Ophthalmology, Genetics, Maternal, and Child Health, School of Medicine, University of Genova, Genova, Italy; 4 Academic Unit of Obstetrics and Gynaecology, IRCCS Ospedale Policlinico San Martino, Genova, Italy; 5 Institut National de la Sante et de la Recherche Medicale Unit 981, Molecular Predictors and New Targets in Oncology, Gustave Roussy, Villejuif, France; 6 Breast Cancer Unit, Medical Oncology Department, Gustave Roussy, Villejuif, France; 7 Unicancer, Paris, France; 8 Department of Medical Oncology, Centre Georges-François Leclerc, Dijon, France; 9 Department of Medical Oncology, Institut Curie, Paris, France; 10 Department of Medical Oncology, Centre Alexis Vautrin, Vandoeuvre Les Nancy, France; 11 Department of Medical Oncology, Institut Curie, Saint-Cloud, France; 12 Department of Surgery, Centre Léon Berard, Lyon, France; 13 Department of Medical Oncology, Institut Jean Godinot, Reims, France; 14 Department of Medical Oncology, Centre Henri Becquerel, Rouen, France; 15 Department of Surgical Oncology, Institut Bergonié, Bordeaux, France; 16 Department of Medical Oncology, Institut de Cancérologie de L’ouest-Paul Papin, Angers, France; 17 Department of Medical Oncology, Dana-Farber Cancer Institute, Harvard Medical School, Boston, Massachusetts

## Abstract

**Importance:**

As life span has increased among patients with cancer, survivorship has become an important component of breast cancer care. Among survivorship concerns, adequate contraceptive counseling is needed for premenopausal patients who are not seeking to become pregnant.

**Objective:**

To examine contraceptive use and chosen methods and to assess factors associated with contraceptive use over time in patients with early breast cancer.

**Design, Setting, and Participants:**

The Cancer Toxicity (CANTO) study was a multicenter nationwide prospective cohort study that enrolled women diagnosed with stage I to stage III breast cancer in France between March 2012 and December 2017. This analysis included 2900 premenopausal women who were 50 years of age or younger at diagnosis. Data were analyzed from July 2020 to July 2022.

**Exposures:**

Contraceptive use and method at diagnosis, shortly after the end of primary treatment (year 1), and during follow-up (year 2).

**Main Outcomes and Measures:**

Contraceptive use and methods were longitudinally evaluated at diagnosis, year 1, and year 2 after breast cancer diagnosis. Multivariable logistic regression models were used to assess the associations of clinical, socioeconomic, treatment, adverse effect, and patient-reported outcome variables with contraceptive use after diagnosis.

**Results:**

A total of 2900 patients (mean [SD] age, 43.1 [5.6] years) were included in the analysis; 2050 of 2894 women (70.8%) received chemotherapy, and 2305 of 2880 women (80.0%) received endocrine therapy. After diagnosis, 1182 of 2625 patients (45.0%) at year 1 and 1553 of 2363 patients (65.7%) at year 2 reported consulting with a gynecologist in the previous year. At diagnosis, 1487 of 2744 patients (54.2%) reported contraceptive use, with most patients (921 of 1470 women [62.7%]) using hormonal methods. The use of contraception significantly decreased after diagnosis (911 of 2342 patients [38.9%] at year 1 and 808 of 1961 patients [41.2%] at year 2; *P* < .001 for trend), when most patients (848 of 900 women [94.2%] at year 1 and 767 of 805 women [95.3%] at year 2) reported use of nonhormonal methods; these methods were primarily reversible mechanical approaches (copper intrauterine devices: 656 of 848 patients [77.4%] at year 1 and 577 of 767 patients [75.2%] at year 2; male condoms: 115 of 848 patients [13.6%] at year 1 and 110 of 767 patients [14.3%] at year 2). In the multivariable model, factors significantly associated with contraceptive use at year 1 included using contraception at diagnosis (adjusted odds ratio [aOR], 4.02; 95% CI, 3.15-5.14), being younger (aOR, 1.09; 95% CI, 1.07-1.13 per decreasing year), having better sexual function (aOR, 1.13; 95% CI, 1.07-1.19 per 10-point increment), having children (aOR, 4.21; 95% CI, 1.80-9.86), reporting the presence of leukorrhea (aOR, 1.32; 95% CI, 1.03-1.70), receiving tamoxifen treatment alone (aOR, 1.39; 95% CI, 1.01-1.92), and consulting with a gynecologist in the previous year (aOR, 1.29; 95% CI, 1.02-1.63). Similar factors were associated with contraceptive use at year 2, with the addition of partnered status (aOR, 1.61; 95% CI, 1.07-2.44).

**Conclusions and Relevance:**

Findings from this study support the importance of raising awareness and improving targeted contraceptive counseling for premenopausal women with early breast cancer.

## Key Points

**Questions:**

How do premenopausal women with early breast cancer use contraception, and what are the chosen methods and factors associated with contraceptive use?

**Findings:**

In this cohort study of 2900 premenopausal women with early breast cancer enrolled in the prospective Cancer Toxicity study, 54.2% of patients reported contraceptive use at the time of diagnosis, with most patients using hormonal methods. Contraceptive use significantly decreased after primary treatment and during follow-up, when most patients reported use of reversible mechanical methods.

**Meaning:**

These findings support the importance of raising awareness and improving targeted contraceptive counseling among premenopausal women with early breast cancer.

## Teaser

This cohort study describes the use and methods of contraception and assesses factors associated with contraceptive use over time among premenopausal women with early breast cancer.

## Introduction

Breast cancer is the most commonly diagnosed cancer in women of reproductive age and has the highest survival rates among solid tumor diseases.^1^ The increase in life span among cancer survivors has created a greater need to address both early and late potential adverse effects of anticancer therapies to maximize quality of life.^2^ Among premenopausal patients, reproductive health is an important component of survivorship care. Although clear guidance exists regarding how to discuss treatment-induced risk of premature ovarian insufficiency and fertility preservation strategies,^3,4^ with well-defined oncofertility models of care,^5^ limited evidence is available regarding how to optimally manage other relevant gynecologic issues in reproductive health, including among women not seeking to become pregnant.^6^ Among these reproductive health issues, contraception is a major concern and represents a frequent reason for gynecologic consultation during and after completion of active anticancer therapies.^7^

In recent years, together with increasing knowledge about the safety of pregnancy among breast cancer survivors,^8^ some concerning signals have emerged suggesting that many pregnancies are unintended and occur in women not interested and not attempting to conceive.^9,10^ This finding partly explains the high rate of induced abortions described among breast cancer survivors.^9^ Moreover, it has been reported that cancer survivors are more likely to use emergency contraception than the general population.^11^ Lack of adequate contraceptive counseling is among the reasons that may explain these findings.^12,13^ Patients may use ineffective methods^13^ or decide not to use contraception based on the mistaken belief that they are infertile during or after the receipt of anticancer therapies.^14^ This misconception is of concern in the breast cancer setting given both the need to extend the duration of potentially teratogenic endocrine therapy agents, such as tamoxifen, for 5 to 10 years and the fact that developing chemotherapy-induced premature ovarian insufficiency does not always translate into infertility because ovarian function can resume up to several years after completion of treatment.^3,4^ Notably, because breast cancer is a hormonally based disease, traditional combined hormonal contraception is contraindicated in women with a history of breast cancer.^15^

Despite the importance of this topic, the existing evidence on contraceptive use among women with cancer has been derived mostly from retrospective studies or surveys with small sample sizes^9,13,16^ and studies including survivors of all cancers that have not focused specifically on those with breast cancer.^11,14,17,18^ Therefore, no adequate evidence exists to guide physicians in performing proper contraceptive counseling for premenopausal women with breast cancer.

In the present cohort study, we aimed to investigate contraceptive use and chosen methods and to assess factors associated with the use of contraception among premenopausal women with early breast cancer. For this purpose, we used data from the Cancer Toxicity (CANTO) cohort, which provided a distinctly informative data set for the assessment of contraceptive use among women with breast cancer.^19^

## Methods

### Data Source

The CANTO study was a multicenter nationwide prospective cohort study that enrolled patients diagnosed with stage I, stage II, or stage III breast cancer in France between March 2012 and December 2017. The current analysis involved 2900 participants from the CANTO cohort, with data analyzed from July 2020 to July 2022. This study was approved by the French ethics committee (Comité de Protection des Personnes Ile de France 7^11^) and the French health authority (Agence Nationale de Sécurité du Médicament et des Produits de Santé). All participants provided written informed consent. The study followed the Strengthening the Reporting of Observational Studies in Epidemiology (STROBE) reporting guideline for cohort studies.^20^

Study procedures were previously described.^19^ Patient assessment at diagnosis included sociodemographic characteristics (age, educational level, monthly household income [with euros converted to US dollars based on the 2021 mean exchange rate, with 1 euro equivalent to 1.183 US dollars], and partnership status), clinicobehavioral characteristics (comorbidities measured by the Charlson Comorbidity Index, body mass index [BMI; calculated as weight in kilograms divided by height in meters squared], self-reported physical activity measured by the 16-item Global Physical Activity Questionnaire,^21^ smoking status, and alcohol consumption), and tumor- and treatment-related characteristics.

Longitudinal evaluations were performed at study entry, year 1 (approximately 1 year after diagnosis and 3-6 months after primary treatment), and year 2 (approximately 2 years after diagnosis and 1 year after primary treatment) and included assessment of several patient-reported outcomes. Posttreatment quality of life was measured using the European Organization for Research and Treatment of Cancer (EORTC) Quality of Life Questionnaire (QLQ)–Core 30 (QLQ-C30), which was designed to measure quality of life among patients with cancer.^22,23,24,25,26^ Patients respond to items about functioning and symptoms using a 4-point Likert scale, with 1 indicating not at all, 2 indicating a little, 3 indicating quite a bit, and 4 indicating very much. A standard scoring algorithm was used to convert responses to a scale of 0 to 100 points, with higher scores for global health status indicating better quality of life, and higher scores for functional scales (physical, emotional, cognitive, social, and role) indicating better function. The scoring procedures followed standards published in the EORTC QLQ-C30 Scoring Manual^25^ and the EORTC QLQ-C30 Manual of Reference Values.^26^ Quality of life associated with breast cancer was measured using the 23-item breast cancer–specific module of the EORTC QLQ (QLQ-BR23). As with the QLQ-C30, patients respond to items about functioning using a 4-point Likert scale, with 1 indicating not at all, 2 indicating a little, 3 indicating quite a bit, and 4 indicating very much. A standard scoring algorithm was used to convert responses to a scale of 0 to 100 points, with higher scores indicating better function. Fatigue was assessed using the fatigue symptom scale of the EORTC QLQ-C30^27,28,29^; a standard scoring algorithm was used to convert responses to a scale of 0 to 100, with higher scores indicating greater severity of fatigue. Scores of 40 points or greater were categorized as severe. Psychological distress was measured using the Hospital Anxiety and Depression Scale,^30^ which comprises 14 items (7 items in the anxiety subscale and 7 items in the depression subscale). Scores for each subscale range from 0 to 21 points, with higher scores indicating a higher likelihood of anxiety or depression. Scores of 0 to 7 points were categorized as no, and scores of 8 points or greater were categorized as possible or yes. Gynecologic symptoms, including vaginal dryness, leukorrhea, and hot flashes, were measured using the Common Terminology Criteria for Adverse Events, version 4.0 (grades 1-5, with grade 1 indicating mild events, grade 2 indicating moderate events, grade 3 indicating severe but not immediately life-threatening events, grade 4 indicating life-threatening events, and grade 5 indicating death). Symptoms of any grade were included. Nurse-assessed adverse effects were measured by the Common Terminology Criteria for Adverse Events (CTCAE), version 4.0.^31^

The use of contraceptive methods was also specifically evaluated by trained clinical research nurses at each time point, with a detailed assessment of the type of contraception used at year 1 and year 2. In addition, during follow-up evaluations at year 1 and year 2, patients were systematically asked about their use of specific health care services over the previous year, including whether they had consulted with a gynecologist. Clinician-reported presence of leukorrhea was used as a proxy for vaginal health.^32^

### Study Cohort

At the time of the present analysis, 9595 patients (among 12 012 total participants enrolled in the CANTO study) who were diagnosed with breast cancer from 2012 to 2017 were included in the cohort, with follow-up available until the second posttreatment visit (year 2). Women who were postmenopausal, were older than 50 years, or had unknown menopausal status at diagnosis were excluded. In addition, we only included women who provided information about contraceptive use at 1 or more time points of interest.

### Study Objectives and Variables of Interest

Contraceptive use (yes vs no) and type of contraceptive method (hormonal vs nonhormonal) were the outcomes of interest. We investigated the use of contraception over time and the factors associated with contraceptive use, including patient-reported outcomes comprising sexual function (measured by EORTC QLQ-BR23 item 14, which asks, “To what extent were you interested in sex?” and item 15, which asks, “To what extent were you sexually active [with or without intercourse]?”)^24^ and clinician-reported outcomes comprising gynecologic symptoms (measured by the CTCAE).^31^

### Statistical Analysis

Cohort characteristics and the use of contraception at year 1 and year 2 were analyzed. Changes in contraceptive use over time were evaluated using the Cochrane-Armitage trend test. An initial working set of variables potentially associated with contraceptive use was chosen based on background clinical knowledge^25,26,27,28,29^ (Table 1). We then used descriptive statistics to summarize cohort characteristics by use of contraception at year 1 and year 2, with χ^2^ tests used for categorical variables and *t* tests used for continuous variables (eTable 1 and eTable 2 in the Supplement). Next, using multivariable logistic regression models, we evaluated the association of outcomes with (1) variables of a priori strong clinical interest and (2) variables that were found to be associated with outcomes in the univariable analyses (ie, were statistically significant at 2-sided *P* < .05). Separate models were constructed for each time point (year 1 and year 2), with adjusted odds ratios (aORs) and 95% CIs calculated for each covariate. All analyses were complete case, and no imputation of missing data was performed.

**Table 1.** Patient Characteristics at Breast Cancer Diagnosis

Characteristic	Patients, No./total No. (%) (N = 2900)
Age at diagnosis, mean (SD), y	43.1 (5.6)
Partnership status	
Not in a relationship	573/2661 (21.5)
In a relationship	2088/2661 (78.5)
Highest educational level	
Primary or high school	1232/2669 (46.2)
College graduate or higher	1437/2669 (53.8)
Monthly household income, $^a^	
<3549	1234/2554 (48.3)
≥3549	1320/2554 (51.7)
Has children	
No	104/2602 (4.0)
Yes	2498/2602 (96.0)
Charlson Comorbidity Index score	
0	2407/2686 (89.6)
≥1	279/2686 (10.4)
BMI, mean (SD)^b^	24.3 (4.9)
Level of physical activity, median (IQR), MET h/wk^c^	15.0 (0.7-42.0)
Smoking status	
Current	729/2856 (25.5)
Former	644/2856 (22.5)
Never	1483/2856 (51.9)
Alcohol consumption	
Daily	265/2812 (9.4)
Less than daily	2547/2812 (90.6)
Cancer stage	
I	1157/2888 (40.1)
II or III	1731/2888 (59.9)
Cancer subtype	
HR^+^/*ERBB2*^−^	1971/2875 (68.6)
HR^+^/*ERBB2*^+^	428/2875 (14.9)
HR^−^/*ERBB2*^+^	124/2875 (4.3)
HR^−^/*ERBB2*^−^	352/2875 (12.2)
Surgical procedure	
Partial mastectomy	1892/2893 (65.4)
Full mastectomy	1001/2893 (34.6)
Axillary procedure	
Sentinel lymph node	1525/2893 (52.7)
Axillary dissection	1368/2893 (47.3)
Chemotherapy	
No	844/2894 (29.2)
Yes	2050/2894 (70.8)
Radiotherapy	
No	222/2889 (7.7)
Yes	2667/2889 (92.3)
Hormonal therapy	
None	575/2880 (20.0)
Tamoxifen treatment alone	1849/2880 (64.2)
Other^d^	456/2880 (15.8)
Anti-*ERBB2* therapy	
No	2416/2891 (83.6)
Yes	475/2891 (16.4)
Anxiety^e^	
No	922/2685 (34.3)
Possible or yes	1763/2685 (65.7)
Depression^e^	
No	2145/2683 (79.9)
Possible or yes	538/2683 (20.1)
Patient-reported quality of life^e^	
Global health status	
Mean (SD)	67.4 (19.0)
Median (IQR)	66.7 (58.3-83.3)
Physical function	
Mean (SD)	93.5 (11.3)
Median (IQR)	100 (93.3-100)
Emotional function	
Mean (SD)	60.7 (24.8)
Median (IQR)	66.7 (41.7-83.3)
Cognitive function	
Mean (SD)	79.3 (23.2)
Median (IQR)	83.3 (66.7-100)
Social function	
Mean (SD)	88.3 (20.0)
Median (IQR)	100 (83.3-100)
Role function	
Mean (SD)	84.7 (22.7)
Median (IQR)	100 (66.7-100)
Severe fatigue^e^	893 (33.1)
Patient-reported breast cancer–specific quality of life^e^	
Body image	
Mean (SD)	86.2 (21.5)
Median (IQR)	100 (83.3-100)
Sexual function	
Mean (SD)	35.0 (26.6)
Median (IQR)	66.7 (33.3-100)
Sexual enjoyment	
Mean (SD)	67.3 (26.9)
Median (IQR)	33.3 (16.7-66.7)
Gynecologic symptoms^e^	
Vaginal dryness	246/2759 (8.9)
Leukorrhea	911/2725 (33.4)
Hot flashes	441/2760 (16.0)

Abbreviations: BMI, body mass index (calculated as weight in kilograms divided by height in meters squared); *ERBB2*, ERB-B2 receptor tyrosine kinase 2 (formerly *HER2* [human epidermal growth factor receptor 2]); HR, hormone receptor; MET, metabolic equivalent of task. The plus and minus signs indicate positive and negative, respectively.

^a^ Monthly household income was converted from euros to US dollars based on the 2021 mean exchange rate (1.183 US dollars = 1 euro), with $3549 equivalent to €3000.

^b^ Data were missing for 14 patients.

^c^ Data were missing for 209 patients.

^d^ Includes treatment with an aromatase inhibitor plus ovarian suppression (n = 105), ovarian suppression alone (n = 17), tamoxifen treatment plus ovarian suppression (n = 53), or tamoxifen treatment and an aromatase inhibitor plus ovarian suppression (n = 281).

^e^ Details on scoring are provided in the Methods section.

In a sensitivity analysis restricting the cohort to women providing data on contraceptive use at all time points, we evaluated the change in contraceptive use over the temporal trajectory. Contraceptive use was categorized as never, persistent (ie, consistent use at diagnosis, year 1, and year 2), discontinuation after diagnosis (ie, discontinuation of use after diagnosis), and start after diagnosis (ie, no use at diagnosis, then initiation of use at year 1 or year 2).

Descriptive statistics were used to compare baseline cohort characteristics across categories, with χ^2^ tests used for categorical variables and *t* tests used for continuous variables. Multinomial logistic regression analysis was used to assess associations between variables collected at diagnosis and contraceptive use in a multivariable setting (with never use serving as the reference category). Additional sensitivity analyses were conducted among (1) patients younger than 45 years at diagnosis and (2) patients who reported being sexually active (defined as an EORTC QLQ-BR23 sexual function score of >0 points, which was used as a proxy for sexual activity).

Statistical analyses were performed using SAS software, version 9.4 (SAS Institute, Inc). Statistical significance was defined as 2-sided *P* < .05.

## Results

### Cohort Characteristics at Diagnosis

**Figure 1.**
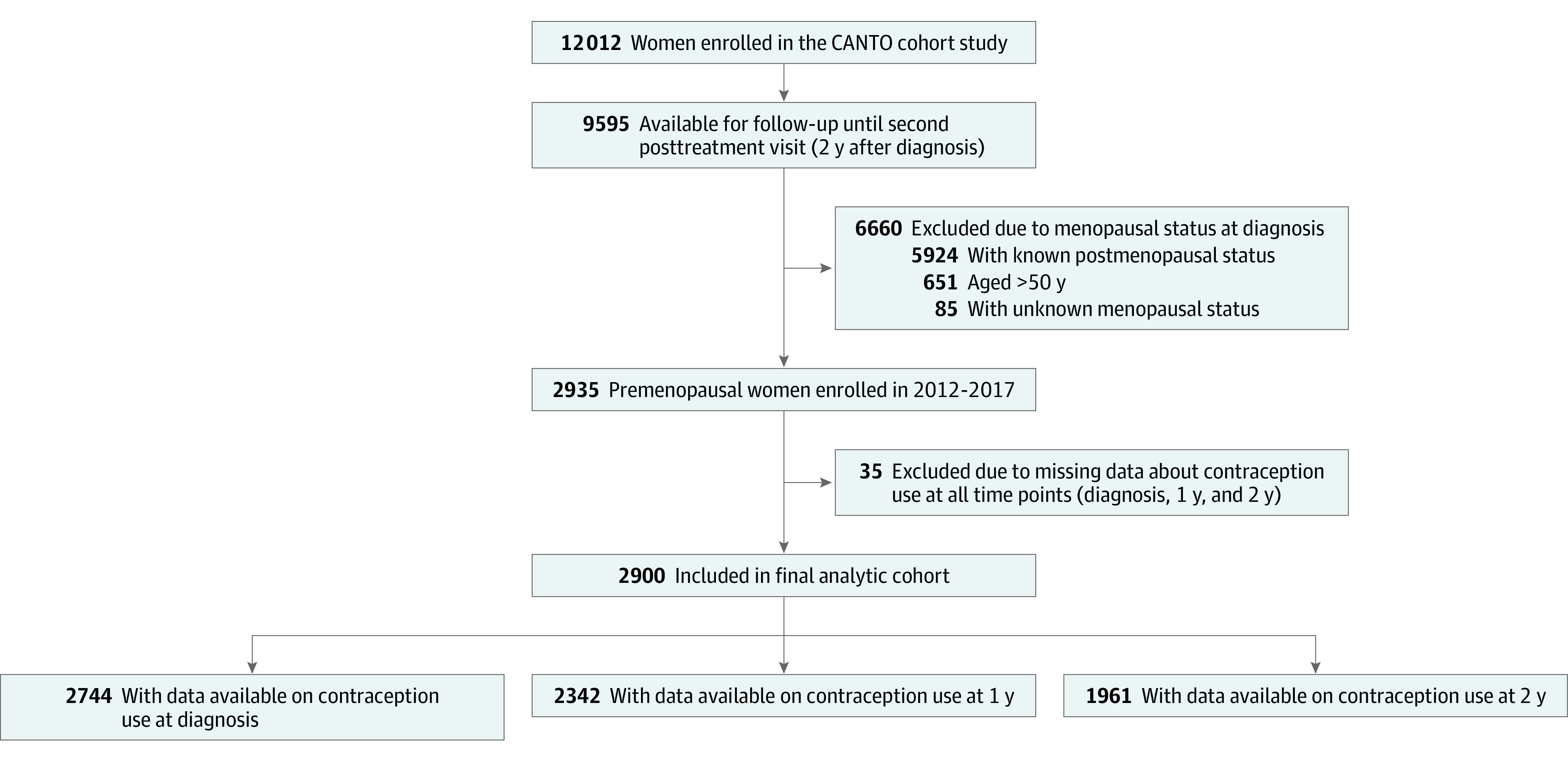
Flowchart of Patient Inclusion CANTO indicates Cancer Toxicity.

A total of 2900 premenopausal women with early breast cancer (mean [SD] age at diagnosis, 43.1 [5.6] years) were included in the present analysis (Figure 1; Table 1). At diagnosis, 2088 of 2661 patients (78.5%) had partnered status, and 2498 of 2602 patients (96.0%) had children. A total of 2050 of 2894 patients (70.8%) had received chemotherapy, and 2305 of 2880 patients (80.0%) had received endocrine therapy. Among 2305 patients who received endocrine therapy, 1849 (80.2%) received tamoxifen treatment alone, and 456 patients (19.8%) received other hormonal therapies that included ovarian function suppression agents. The mean (SD) score for EORTC QLQ-BR23 sexual function was 35.0 (26.6) points.

### Contraceptive Use, Methods, and Changes Over Time

Overall, 1487 of 2744 women (54.2%) reported using contraception at diagnosis, 911 of 2342 women (38.9%) reported using contraception at year 1, and 808 of 1961 women (41.2%) reported using contraception at year 2 (*P* < .001 for trend) (Table 2). Hormonal methods were used among 921 of 1470 women (62.7%) at diagnosis, 52 of 900 patients (5.8%) at year 1, and 38 of 805 patients (4.7%) at year 2. Most women reported use of nonhormonal methods at year 1 (848 of 900 patients [94.2%]) and year 2 (767 of 805 patients [95.3%]), which mostly comprised reversible mechanical methods (772 of 848 patients [91.0%] at year 1 and 688 of 767 patients [89.7%] at year 2; some patients reported using >1 type of contraceptive method), including copper intrauterine devices (IUDs; 656 of 848 patients [77.4%] at year 1 and 577 of 767 patients [75.2%] at year 2) and male condoms (115 of 848 patients [13.6%] at year 1 and 110 of 767 patients [14.3%] at year 2). A total of 36 of 848 women (4.2%) at year 1 and 28 of 767 women (3.7%) at year 2 reported using nonreversible procedures (Essure, hysterectomy, ovariectomy, salpingectomy, or both ovariectomy and salpingectomy; some patients reported using >1 type of contraceptive method). In addition, during follow-up, 1182 of 2625 patients (45.0%) at the year 1 visit and 1553 of 2363 patients (65.7%) at the year 2 visit reported consulting with a gynecologist in the previous year.

**Table 2.** Use and Type of Contraception at Diagnosis, Year 1, and Year 2

Type of contraception	Patients, No./total No. (%)^a^		
	Contraceptive use at diagnosis (n = 2744)	Contraceptive use at 1 y (n = 2342)	Contraceptive use at 2 y (n = 1961)
None^b^	1257/2744 (45.8)	1431/2342 (61.1)	1153/1961 (58.8)
Any^c^	1487/2744 (54.2)	911/2342 (38.9)	808/1961 (41.2)
Known method	1470/1487 (98.9)	900/911 (98.8)	805/808 (99.6)
Hormonal	921/1470 (62.7)	52/900 (5.8)	38/805 (4.7)
Hormonal IUD	NA	34/52 (65.4)	24/38 (63.2)
Pill	NA	9/52 (17.3)	2/38 (5.3)
Implant	NA	8/52 (15.4)	11/38 (28.9)
Nonhormonal	549/1470 (37.3)	848/900 (94.2)	767/805 (95.3)
Copper IUD	NA	656/848 (77.4)	577/767 (75.2)
Male condom	NA	115/848 (13.6)	110/767 (14.3)
Tubal ligation	NA	29/848 (3.4)	39/767 (5.1)
Essure	NA	23/848 (2.7)	14/767 (1.8)
Ovariectomy and salpingectomy	NA	2/848 (0.2)	6/767 (0.8)
Ovariectomy	NA	6/848 (0.7)	4/767 (0.5)
Salpingectomy	NA	3/848 (0.4)	2/767 (0.3)
Spermicide	NA	3/848 (0.4)	3/767 (0.4)
Hysterectomy	NA	2/848 (0.2)	2/767 (0.3)
Vasectomy	NA	2/848 (0.2)	1/767 (0.1)
Abstinence	NA	1/848 (0.1)	1/767 (0.1)
Diaphragm	NA	1/848 (0.1)	1/767 (0.1)
Other^d^	NA	5/848 (0.6)	7/767 (0.9)
Unknown method^e^	17/1487 (1.1)	11/911 (1.3)	3/808 (0.4)

Abbreviations: IUD, intrauterine device; NA, not available.

^a^ Among 2900 total patients in the cohort, data on contraception were missing for 156 patients (5.4%) at diagnosis, 558 patients (19.2%) at year 1, and 939 patients (32.4%) at year 2. A total of 13 patients exited the study due to recurrence or death occurring between diagnosis and year 1, and 82 patients exited due to recurrence or death occurring between year 1 and year 2. In addition, 11 patients reported pregnancy at year 1, and 13 patients reported pregnancy at year 2.

^b^ Proportions of the total cohort (N = 2900) who were not using contraception were 43.3% at diagnosis, 49.3% at year 1, and 39.8% at year 2.

^c^ Proportions of the total cohort (N = 2900) who were using any type of contraception were 51.3% at diagnosis, 31.4% at year 1, and 27.9% at year 2.

^d^ No information was available regarding the specific nonhormonal method used.

^e^ Includes hormonal as well as nonhormonal methods; however, no further information was available regarding the specific method used.

### Factors Associated With Contraceptive Use

#### Contraceptive Use at Year 1

**Figure 2.**
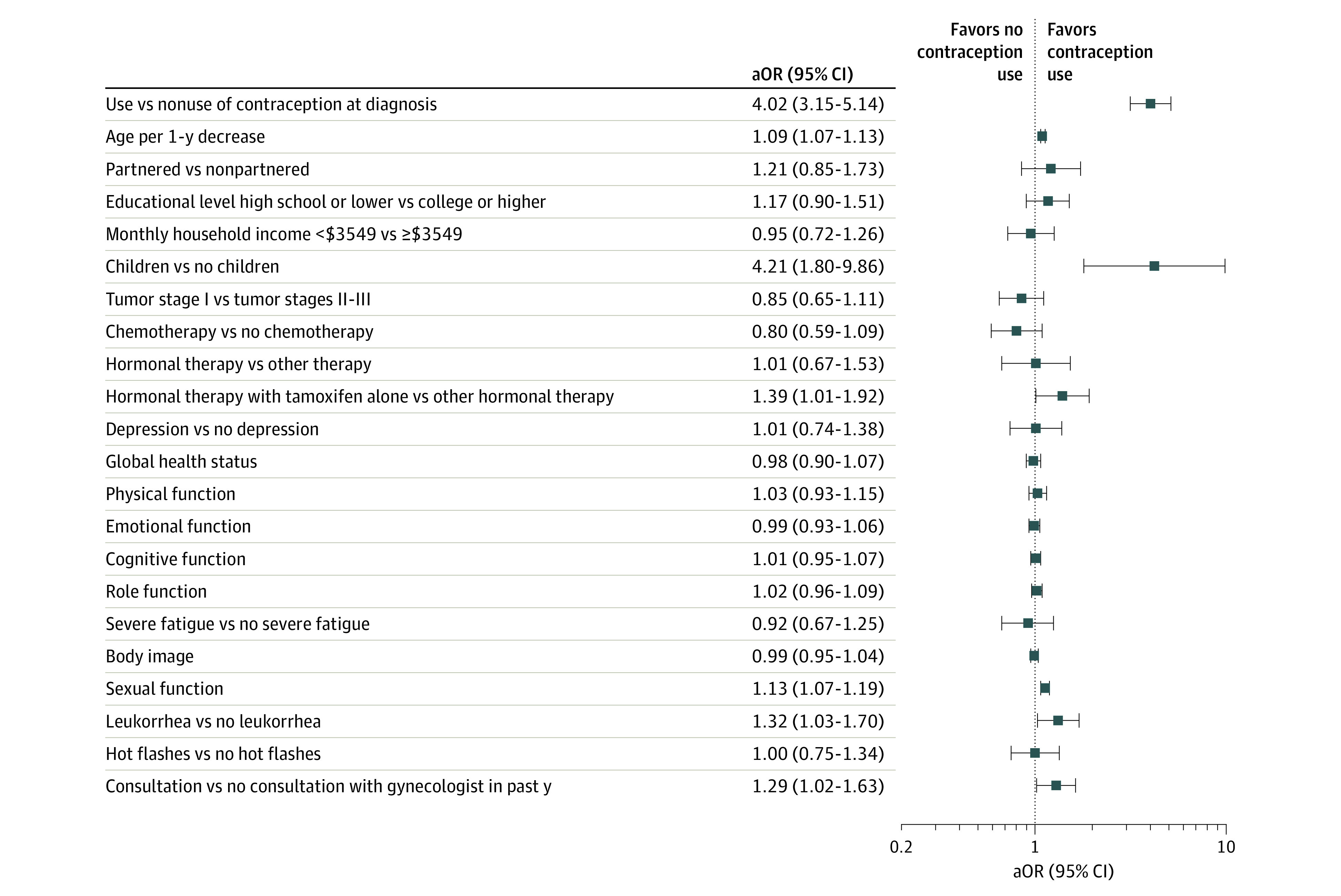
Factors Associated With Contraceptive Use at Year 1 Results of multivariable logistic regression analysis adjusting for all factors presented in the forest plot. Estimates for continuous variables represent changes in the odds of the outcome for each 10-point increment in the variable value. Monthly household income was converted from euros to US dollars based on the 2021 mean exchange rate (1.183 US dollars = 1 euro), with $3549 equivalent to €3000. aOR indicates adjusted odds ratio.

In univariate analyses, at the year 1 follow-up visit, patients reporting contraceptive use (n = 911) vs no contraceptive use (n = 1431) were more likely to be younger (mean [SD] age, 41.6 [5.8] years vs 43.9 [5.2] years), have higher socioeconomic status (monthly household income ≥$3549 [equivalent to €3000]: 470 of 822 women [57.2%] vs 631 of 1273 women [49.6%]), have partnered status (721 of 855 women [84.3%] vs 995 of 1324 women [75.2%]), have children (838 of 852 women [98.4%] vs 1178 of 1249 women [94.3%]), more frequently report the presence of leukorrhea (330 of 911 women [36.2%] vs 394 of 1431 women [27.5%]), and less frequently report experiencing hot flashes (657 of 911 women [72.1%] vs 1118 of 1431 women [78.1%]) (eTable 1 in the Supplement). Patients reporting use vs nonuse of contraception also had higher scores on several patient-reported outcome domains of the EORTC QLQ-BR23 and QLQ-C30, including body image (mean [SD], 67.4 [30.8] points vs 62.7 [32.2] points), sexual function (mean [SD], 42.1 [23.1] points vs 31.2 [25.0] points), sexual enjoyment (mean [SD], 61.9 [26.0] points vs 59.0 [27.4] points), global health status (mean [SD], 69.1 [18.0] points vs 66.3 [18.5] points), physical function (mean [SD], 87.6 [13.2] points vs 85.1 [14.6] points), emotional function (mean [SD], 70.9 [24.8] points vs 67.5 [25.6] points), cognitive function (mean [SD], 73.4 [26.5] points vs 77.0 [24.4] points), role function (mean [SD], 76.2 [23.7] points vs 79.0 [24.2] points), and social function (mean [SD], 81.3 [24.5] points vs 77.9 [26.4] points). In the final multivariable model, factors significantly associated with contraceptive use at year 1 included using contraception at diagnosis (aOR, 4.02; 95% CI, 3.15-5.14), being younger (aOR, 1.09; 95% CI, 1.07-1.13 per each decreasing year), having better sexual function (aOR, 1.13; 95% CI, 1.07-1.19 per 10-point increment), having children (aOR, 4.21; 95% CI, 1.80-9.86), reporting the presence of leukorrhea (aOR, 1.32; 95% CI, 1.03-1.70), receiving tamoxifen treatment alone (aOR, 1.39; 95% CI, 1.01-1.92), and consulting with a gynecologist in the previous year (aOR, 1.29; 95% CI, 1.02-1.63) (Figure 2).

#### Contraceptive Use at Year 2

**Figure 3.**
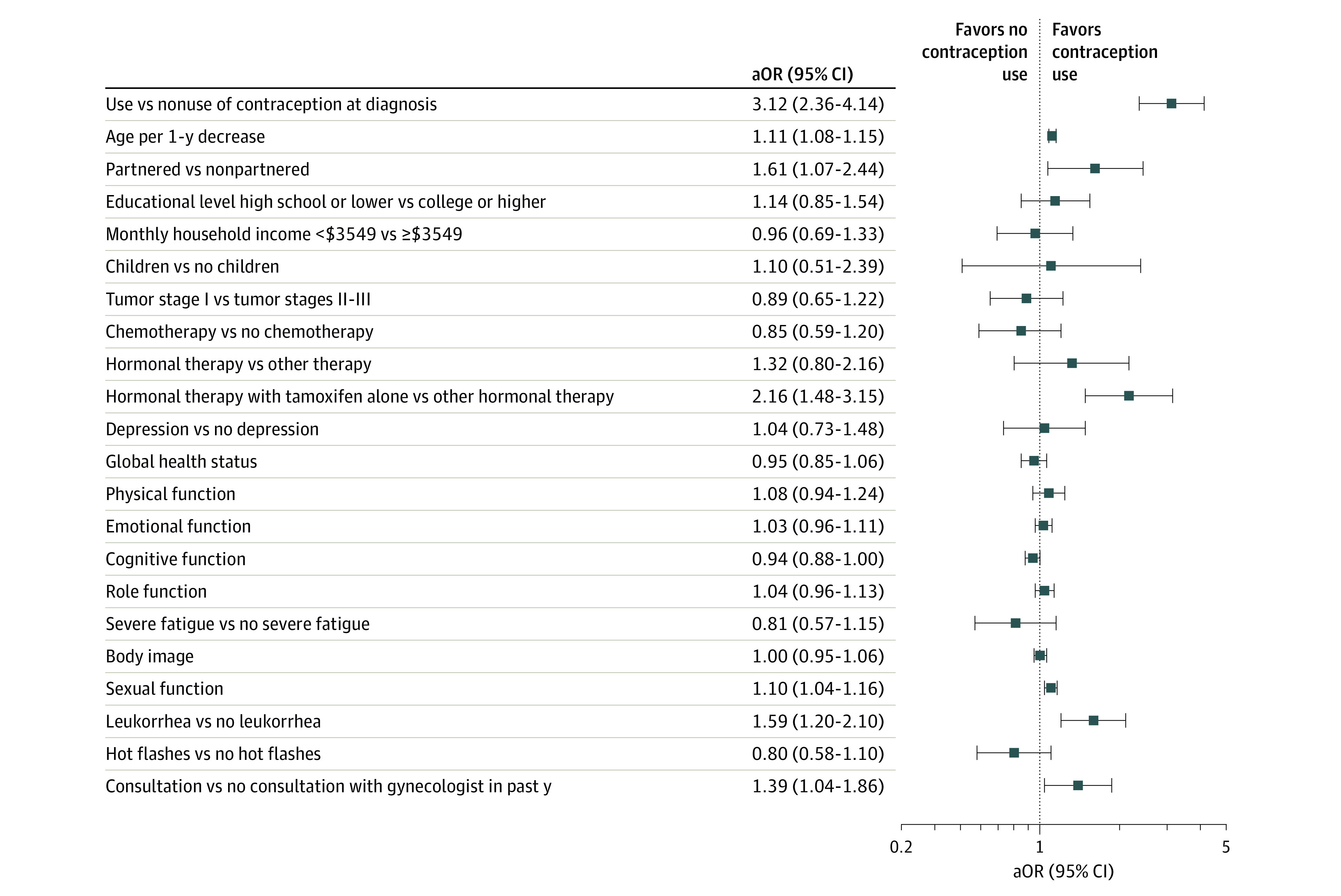
Factors Associated With Contraceptive Use at Year 2 Results of multivariable logistic regression analysis adjusting for all factors presented in the forest plot. Estimates for continuous variables represent changes in the odds of the outcome for each 10-point increment in the variable value. Monthly household income was converted from euros to US dollars based on the 2021 mean exchange rate (1.183 US dollars = 1 euro), with $3549 equivalent to €3000. aOR indicates adjusted odds ratio.

Results of the univariate analysis at year 2 were consistent with those at year 1. For example, at the year 2 follow-up visit, patients reporting contraceptive use (n = 808) vs no contraceptive use (n = 1153) were more likely to be younger (mean [SD] age at diagnosis, 41.3 [5.8] years vs 44.2 [5.0] years), have higher socioeconomic status (monthly household income ≥$3549 [equivalent to €3000]: 425 of 743 women [57.2%] vs 548 of 1048 women [52.3%]), have partnered status (653 of 772 women [84.6%] vs 813 of 1087 women [74.8%]), more frequently report the presence of leukorrhea (374 of 808 women [46.3%] vs 361 of 1153 women [31.3%]), less frequently report experiencing hot flashes (552 of 808 women [68.3%] vs 883 of 1153 women [76.6%]) and have higher EORTC QLQ-BR23 and QLQ-C30 posttreatment outcome scores for body image (mean [SD], 75.1 [28.2] points vs 70.0 [30.6] points), sexual function (mean [SD], 44.3 [23.1] points vs 32.7 [26.5] points), global health status (mean [SD], 68.7 [17.8] points vs 65.7 [18.6] points), physical function (mean [SD], 89.7 [12.0] points vs 85.8 [14.7] points), emotional function (mean [SD], 70.1 [24.2] points vs 66.3 [26.0] points), role function (mean [SD], 81.2 [23.5] points vs 84.6 [20.9] points), and social function (mean [SD], 85.6 [20.9] points vs 82.1 [24.4] points) (eTable 2 in the Supplement). In the multivariable models, similar factors were significantly associated with contraceptive use at year 2 (eg, using contraception at diagnosis: aOR, 3.12 [95% CI, 2.36-4.14]; being younger: aOR, 1.11 [95% CI, 1.08-1.15]; having better sexual function: aOR, 1.10 [95% CI, 1.04-1.16]; reporting the presence of leukorrhea: aOR, 1.59 [95% CI, 1.20-2.10]; receiving tamoxifen treatment alone: aOR, 2.16 [95% CI, 1.48-3.15]; consulting with a gynecologist in the previous year: aOR, 1.39 [95% CI, 1.04-1.86]) (Figure 3). In addition, partnered status (aOR, 1.61; 95% CI, 1.07-2.44) was also significantly associated with the use of contraception at year 2 (full models are available in eTable 3 in the Supplement).

### Sensitivity Analyses

Among 1640 women who provided data on contraceptive use at all time points, 476 patients (29.0%) reported never using contraception, 439 (26.8%) reported persistently using contraception (at diagnosis and during follow-up), 500 (30.5%) reported discontinuing use of contraception after diagnosis, and 225 (13.7%) reported starting use of contraception after diagnosis. Results consistent with the primary analysis were observed (eTable 4 and eTable 5 in the Supplement). Results of the sensitivity analyses of patients younger than 45 years at diagnosis (1507 women) and patients reporting they were sexually active (1877 women at year 1 and 1574 women at year 2) were also consistent with the primary analysis (eTable 6 and eTable 7 in the Supplement).

## Discussion

This analysis of the CANTO cohort study used prospectively collected data at different time points to provide distinct insights on contraceptive use and factors associated with contraceptive use over time among premenopausal women with early breast cancer. Overall, 54.2% of patients reported contraceptive use at the time of breast cancer diagnosis, but a significant decrease in use was observed during primary treatment (year 1) and follow-up (year 2). Although most women were receiving hormonal contraception at diagnosis, almost all patients transitioned to using nonhormonal strategies during follow-up; of those, most women (91.0% at year 1 and 89.7% at year 2) used reversible mechanical methods. Using contraception at diagnosis, being younger, having children, receiving tamoxifen treatment alone, having better sexual function, reporting the presence of physiological leukorrhea, and consulting with a gynecologist in the previous year were associated with higher odds of contraceptive use after diagnosis, with partnered status also significantly associated with higher odds of contraceptive use at follow-up year 2.

In the present CANTO analysis, 54.2% of women were using contraception at diagnosis, which is similar to rates observed among the healthy population (approximately 60%).^33^ During breast cancer follow-up, this rate decreased to 38.9% at year 1 and 41.2% at year 2. Cancer-related therapy, changes in menopausal status, and suboptimal sexual health among breast cancer survivors may have been associated with the observed rates. Factors associated with sexual health, including patient-reported sexual function (ie, interest in sex and extent of sexual activity with or without intercourse, as measured by items 14 and 15 on the EORTC QLQ-BR23) and clinician-reported presence of physiological leukorrhea (used as a proxy for physiological vaginal health,^32^ as measured by the CTCAE) had consistent positive associations with contraceptive use. These 2 factors were persistently lower among women who did not report contraceptive use compared with those who did, without apparent improvement during follow-up. Chemotherapy and adjuvant endocrine therapy for the treatment of breast cancer can have major consequences for patients’ sexual lives and have been associated with important adverse effects, including decreased libido and vaginal dryness.^2^ Patients with cancer often report low rates of overall sexual satisfaction, and these problems are not always properly addressed.^34^ Considering the observed positive association between contraceptive use and better sexual health, regular sexual activity may motivate women to be more active regarding contraception. However, the use of safe and reliable contraception itself may play a role in facilitating the resumption of a regular and satisfactory sexual life. Thus, it is important that contraceptive counseling is included as part of gynecologic consultation with young breast cancer survivors.

Lack of adequate contraceptive counseling may be associated with suboptimal use among premenopausal women with breast cancer.^12,13^ As a consequence, patients may more frequently require emergency contraception^11^ or choose ineffective methods.^13^ Moreover, an inaccurate perception of infertility due to concurrent or previous exposure to anticancer therapies or misconceptions and fear of being exposed to additional pharmacological agents in the context of their breast cancer history have also been reported by breast cancer survivors.^14^ Therefore, systematically offering gynecologic consultations before initiating breast cancer treatment has been proposed as a way to overcome this barrier.^35^ In the CANTO study, participation in a gynecologic consultation during survivorship follow-up care was associated with a higher likelihood of contraceptive use. Based on current standards, building a well-functioning network between oncological and gynecologic units is necessary to optimize fertility care for all women of reproductive age with newly diagnosed cancer.^3,4^ However, additional needs beyond fertility-related issues exist for these patients during follow-up care.^7^ Taken together, these data support the clinical utility of promoting a more structured referral network that links oncologists and gynecologists, which may ensure not only access to fertility preservation strategies but also provide comprehensive long-term follow-up care that includes optimal contraceptive counseling.

Specialized contraceptive counseling is also important to selecting the most appropriate and reliable method,^18,36^ a particularly sensitive issue among those with hormonally based tumors such as breast cancer. With the exception of a 2-week controlled ovarian stimulation for oocyte or embryo cryopreservation before starting chemotherapy,^37^ exposure to hormonal-based therapies may increase the risk of recurrence.^38^ Thus, these therapies are contraindicated in women with breast cancer. Therefore, the ideal contraceptive methods in this cohort were nonhormonal approaches with good reliability and tolerability. Among those approaches, other than barrier methods (ie, male condoms), the copper IUD can be used during and after receipt of anticancer treatments.^39,40,41^

Notably, approximately 90% of patients chose to use reversible mechanical methods after diagnosis, with male condoms being the most commonly used despite the low effectiveness of this method. The percentage of women who opted for permanent contraceptive procedures (approximately 4%) was lower than the rate expected in the general population.^33^ A possible explanation is the high interest in future childbearing among young women with breast cancer.^8^ Long-term follow-up of participants in the CANTO cohort will provide further data on this topic. With regard to the copper IUD, which is a long-acting reversible nonhormonal method with high reliability in preventing unwanted pregnancies, the low acceptance rates in our study may be explained by the desire to avoid more medicalization or by the cultural resistance to its use, as observed in the general population.^42^

Special considerations and attention are needed for women with hormone receptor–positive breast cancer. Our data revealed that among women receiving adjuvant endocrine therapy, use of tamoxifen treatment alone was associated with higher contraceptive use. Among women receiving tamoxifen, off-label use of the levonorgestrel-releasing IUD has been suggested as an alternative contraceptive method because no evidence of worsening cancer outcomes has been reported.^43^ However, this approach has been used more frequently as a means to protect the endometrium during tamoxifen treatment rather than as a contraceptive method, and some evidence of a potential increased risk of breast cancer recurrence has been observed.^44^ Hence, the risk-benefit ratio of the use of a levonorgestrel-releasing IUD in this setting remains uncertain.^15^

Pharmacological ovarian suppression with gonadotropin-releasing hormone agonists is currently the standard of care as adjuvant endocrine therapy for the treatment of most patients.^45^ Administration of gonadotropin-releasing hormone agonists produces a reversible hypogonadotropic hypogonadism that has a potential contraceptive effect after the initial flare up with the first injection; however, taking into account the possible inadequate ovarian suppression obtained with gonadotropin-releasing hormone agonists, these agents are not licensed or listed as a contraceptive method.^39^ Therefore, the recommendation for the use of barrier methods or a copper IUD remains, including for premenopausal women with hormone receptor–positive breast cancer receiving pharmacological ovarian suppression as adjuvant endocrine therapy. Raising awareness about this issue is relevant given recent changes in clinical practice regarding the use of adjuvant ovarian suppression.^45^ It is possible that more frequent use of these agents will also further increase the nonuse of adequate contraception among premenopausal women receiving adjuvant hormonal therapy. As observed among women without a history of cancer,^46^ these data highlight the importance of specialized comprehensive contraceptive counseling to aid in the selection of reliable methods and decrease the risk of unintended pregnancies.

### Limitations

This study has limitations. Despite being a multicenter study, CANTO reflects the practice of a single country. Longer follow-up of patients enrolled in the CANTO study will provide additional data on contraceptive use over time and further information about the impact of other important factors, including posttreatment pregnancies. Data on pregnancy intentions and attempts were not collected. In addition, the CANTO study did not collect information on sexual orientation, and a sexual function score of greater than 0 points on the EORTC QLQ-BR23 was used as a proxy for sexual activity. Some standards of practice have changed since the data collection period, including increased use of gonadotropin-releasing hormone agonists as part of adjuvant endocrine therapy for premenopausal women. All analyses were complete case, and no imputation of missing data was performed. Moreover, as expected, some attrition over time occurred.

## Conclusions

This large analysis of the CANTO cohort study provided insight into contraceptive use and factors associated with contraceptive use over time among premenopausal women with early breast cancer. These findings support the need to raise awareness and improve targeted contraceptive counseling for these patients at both diagnosis and follow-up. The study also highlights several factors important to contraceptive decision-making. Among those, consulting with a gynecologist after diagnosis was associated with greater contraceptive use, suggesting a need to promote long-term follow-up care by oncofertility units, which could not only provide access to fertility preservation strategies but also promote reproductive health care and contraceptive counseling in the expanding and vulnerable population of premenopausal breast cancer survivors.

## Notes

Supplement. **eTable 1.** Patient Characteristics by Use of Contraception at Year 1 **eTable 2.** Patient Characteristics by Use of Contraception at Year 2 **eTable 3.** Multivariable Logistic Regression Models of Factors Associated With Use of Contraception at Year 1 and Year 2 **eTable 4.** Patient Characteristics by Use of Contraception Over Time (n = 1640) **eTable 5.** Multivariable Multinomial Logistic Regression Models of Use of Contraception Over Time (n = 1640) **eTable 6.** Sensitivity Analysis Among Patients Younger Than 45 Years at Diagnosis: Multivariable Logistic Regression Models of Factors Associated With Use of Contraception at Year 1 and Year 2 **eTable 7.** Sensitivity Analysis Among Patients Who Reported They Were Sexually Active (Defined as Sexual Function Score >0 Points): Multivariable Logistic Regression Models of Factors Associated With Use of Contraception at Year 1 and Year 2
