# Supplementary material for: Contraceptive Use in Premenopausal Women With Early Breast Cancer
Source: JAMA Netw Open. 2022 Sep 23;5(9):e2233137. doi: 10.1001/jamanetworkopen.2022.33137 (PMC9508662; doi:10.1001/jamanetworkopen.2022.33137)
Supplement: Supplement. — eTable 1. Patient Characteristics by Use of Contraception at Year 1 eTable 2. Patient Characteristics by Use of Contraception at Year 2 eTable 3. Multivariable Logistic Regression Models of Factors Associated With Use of Contraception at Year 1 and Year 2 eTable 4. Patient Characteristics by Use of Contraception Over Time (n = 1640) eTable 5. Multivariable Multinomial Logistic Regression Models of Use of Contraception Over Time (n = 1640) eTable 6. Sensitivity Analysis Among Patients Younger Than 45 Years at Diagnosis: Multivariable Logistic Regression Models of Factors Associated With Use of Contraception at Year 1 and Year 2 eTable 7. Sensitivity Analysis Among Patients Who Reported They Were Sexually Active (Defined as Sexual Function Score >0 Points): Multivariable Logistic Regression Models of Factors Associated With Use of Contraception at Year 1 and Year 2 [file jamanetwopen-e2233137-s001.pdf]

Supplementary Online Content

Lambertini M, Massarotti C, Havas J, et al. Contraceptive use in premenopausal women with early breast cancer. *JAMA Netw Open*. 2022;5(9):e2233137. doi:10.1001/jamanetworkopen.2022.33137

eTable 1. Patient Characteristics by Use of Contraception at Year 1

eTable 2. Patient Characteristics by Use of Contraception at Year 2

eTable 3. Multivariable Logistic Regression Models of Factors Associated With Use of Contraception at Year 1 and Year 2

eTable 4. Patient Characteristics by Use of Contraception Over Time (n = 1640)

eTable 5. Multivariable Multinomial Logistic Regression Models of Use of Contraception Over Time (n = 1640)

eTable 6. Sensitivity Analysis Among Patients Younger Than 45 Years at Diagnosis: Multivariable Logistic Regression Models of Factors Associated With Use of Contraception at Year 1 and Year 2

eTable 7. Sensitivity Analysis Among Patients Who Reported They Were Sexually Active (Defined as Sexual Function Score >0 Points): Multivariable Logistic Regression Models of Factors Associated With Use of Contraception at Year 1 and Year 2

This supplementary material has been provided by the authors to give readers additional information about their work.

© 2022 Lambertini M et al. *JAMA Network Open*.

eTable 1. Patient Characteristics by Use of Contraception at Year 1

	By use of contraception at year 1		
**N (%)**	**Yes<br>N= 911**	**No<br>N= 1431**	**p***
**Total**			
**Age at diagnosis, years**			<.0001
Mean (SD)	41.6 (5.8)	43.9 (5.2)	
Missing	0	0	
**Marital Status**			<.0001
Not in a relationship	134 (15.7)	329 (24.8)	
In a relationship	721 (84.3)	995 (75.2)	
Missing	56	107	
**Highest education level**			0.0005
Primary or high school	349 (40.5)	638 (48.0)	
College graduate or higher	513 (59.5)	691 (52.0)	
Missing	49	102	
**Monthly household income**			0.0007
<3,000 Euro	352 (42.8)	642 (50.4)	
≥3,000 Euro	470 (57.2)	631 (49.6)	
Missing	89	158	
**Having children**			<.0001
No	14 (1.6)	71 (5.7)	
Yes	838 (98.4)	1178 (94.3)	
Missing	59	182	
**Charlson Comorbidity Index**			0.9232
0	48	105	
1+	773 (89.6)	1186 (89.4)	
Missing	90 (10.4)	140 (10.6)	
**Body Mass Index, Kg/m^2**			0.0184
Mean (SD)	24.0 (4.5)	24.5 (5.1)	
Missing	5	5	
**Level of physical activity, MET-h/week**			0.0338
Median (Q1-Q3)	15.0 (2.0–41.0)	16.0 (0.0–42.0)	
Missing	48	91	
**Smoking behavior**			0.7323
Current smoker	226 (25.0)	350 (24.9)	
Former smoker	219 (24.3)	322 (22.9)	
Never smoker	458 (50.7)	732 (52.1)	
Missing	8	27	

© 2022 Lambertini M et al. *JAMA Network Open*.

**Alcohol frequency consumption**			0.7156
Daily	806 (90.6)	1249 (90.1)	
Less-than-daily	84 (9.4)	137 (9.9)	
Missing	21	45	
**Tumor stage**			0.0066
I	393 (43.2)	537 (37.6)	
II/III	517 (56.8)	893 (62.4)	
Missing	1	1	
**Tumor subtype**			0.7188
HR+/HER2-	638 (70.4)	977 (68.7)	
HR+/HER2+	129 (14.2)	215 (15.1)	
HR-/HER2+	41 (4.5)	60 (4.2)	
HR-/HER2-	98 (10.8)	171 (12.0)	
Missing	5	8	
**Breast surgery**			0.5473
Partial surgery	593 (65.1)	914 (63.9)	
Mastectomy	318 (34.9)	517 (36.1)	
Missing	0	0	
**Axillary surgery**			0.0135
Sentinel lymph node	506 (55.5)	720 (50.3)	
Axillary dissection	405 (44.5)	711 (49.7)	
Missing	0	0	
**Chemotherapy**			0.0103
No	292 (32.1)	388 (27.1)	
Yes	619 (67.9)	1043 (72.9)	
Missing	0	0	
**Radiation therapy**			0.7589
No	65 (7.1)	107 (7.5)	
Yes	845 (92.9)	1323 (92.5)	
Missing	1	1	
**Hormonal Therapy**			0.0001
No	160 (17.6)	280 (19.6)	
Tamoxifen alone	636 (70.0)	889 (62.3)	
Other hormonal therapy	113 (12.4)	259 (18.1)	
Missing	2	3	
**Anti-HER2 therapy**			0.5413
No	763 (83.8)	1186 (82.9)	
Yes	147 (16.2)	245 (17.1)	
Missing	1	0	
**Anxiety**			0.5788
Non-case (score 0-7)	297 (34.5)	446 (33.3)	
Doubtful or case (score ≥8)	565 (65.5)	893 (66.7)	

© 2022 Lambertini M et al. *JAMA Network Open*.

Missing	49	92	
**Depression**			0.0569
Non-case (score 0-7)	1060 (79.2)	710 (82.5)	
Doubtful or case (score ≥8)	279 (20.8)	151 (17.5)	
Missing	92	50	
**Patient-reported Quality of Life (EORTC QLQ-C30), Mean (SD)****			
Global Health Status	69.1 (18.0)	66.3 (18.5)	0.0007
Physical Function	87.6 (13.2)	85.1 (14.6)	<.0001
Emotional Function	70.9 (24.8)	67.5 (25.6)	0.0026
Cognitive Function	73.4 (26.5)	77.0 (24.4)	0.0015
Social Function	81.3 (24.5)	77.9 (26.4)	0.0028
Role Function	76.2 (25.7)	79.0 (24.2)	0.0135
**Severe Fatigue (EORTC QLQ-C30)****	320 (39.4)	571 (44.7)	0.0158
**Patient-reported Quality of Life (EORTC QLQ-BR23), Mean (SD)****			
Body Image	67.4 (30.8)	62.7 (32.2)	0.0010
Sexual Function	42.1 (23.1)	31.2 (25.0)	<.0001
Sexual Enjoyment	61.9 (26.0)	59.0 (27.4)	0.0328
**Gynecological symptoms (CTCAE v4.0 any grade)****			
Vaginal dryness	327 (35.9)	482 (33.7)	0.2724
Leucorrhea	330 (36.2)	394 (27.5)	<.0001
Hot Flashes	657 (72.1)	1118 (78.1)	0.0009
**Consultation with a gynecologist over the previous year (between diagnosis and year-1 visit)****			
No	433 (49.0)	794 (56.8)	0.0003
Yes	451 (51.0)	604 (43.2)	
Missing	27	33	

*Chi square test for categorical and t-test for continuous variables. **Collected at year-1 follow-up visit. All the other presented characteristics were collected at breast cancer diagnosis. SD=Standard Deviation; MET=Metabolic Equivalent of Task; HR=Hormone receptor; HER2=Human Epidermal Growth Factor Receptor 2; EORTC QLQ=European Organisation for Research and Treatment of Cancer Quality of Life Questionnaire; CTCAE=Common Terminology Criteria for Adverse Events.

© 2022 Lambertini M et al. *JAMA Network Open*.

eTable 2. Patient Characteristics by Use of Contraception at Year 2

	By use of contraception at year 2		
**N (%)**	**Yes N= 808**	**No N= 1153**	**p***
**Total**			
**Age at diagnosis, years**			<.0001
Mean (SD)	41.3 (5.8)	44.2 (5.0)	
Missing	0	0	
**Marital Status**			<.0001
Not in a relationship	119 (15.4)	274 (25.2)	
In a relationship	653 (84.6)	813 (74.8)	
Missing	36	66	
**Highest education level**			0.0005
Primary or high school	298 (38.3)	509 (46.4)	
College graduate or higher	480 (61.7)	587 (53.6)	
Missing	30	57	
**Monthly household income**			0.0398
<3,000 Euro	318 (42.8)	500 (47.7)	
≥3,000 Euro	425 (57.2)	548 (52.3)	
Missing	65	105	
**Having children**			0.1747
No	23 (3.0)	43 (4.3)	
Yes	734 (97.0)	962 (95.7)	
Missing	51	148	
**Charlson Comorbidity Index**			0.7101
0	697 (90.1)	948 (89.5)	
1+	77 (9.9)	111 (10.5)	
Missing	34	94	
**Body Mass Index, Kg/m^2**			0.0008
Mean (SD)	23.8 (4.5)	24.6 (5.0)	
Missing	3	2	
**Level of physical activity, MET-h/week**			0.0882
Median (Q1-Q3)	14.0 (3.0–38.0)	16.0 (2.0–45.3)	
Missing	29	52	
**Smoking behavior**			0.7661
Current smoker	184 (23.1)	270 (23.9)	
Former smoker	195 (24.4)	261 (23.1)	
Never smoker	419 (52.5)	601 (53.1)	
Missing	10	21	
**Alcohol frequency consumption**			0.6186

© 2022 Lambertini M et al. *JAMA Network Open*.

Daily	71 (9.0)	94 (8.4)	
Less-than-daily	714 (91.0)	1026 (91.6)	
Missing	23	33	
**Tumor stage**			0.0109
I	362 (44.9)	451 (39.1)	
II/III	444 (55.1)	701 (60.9)	
Missing	2	1	
**Tumor subtype**			0.3732
HR+/HER2-	582 (72.4)	813 (71.0)	
HR+/HER2+	102 (12.7)	176 (15.4)	
HR-/HER2+	34 (4.2)	46 (4.0)	
HR-/HER2-	86 (10.7)	110 (9.6)	
Missing	4	8	
**Breast surgery**			0.9633
Partial surgery	532 (65.8)	758 (65.7)	
Mastectomy	276 (34.2)	395 (34.3)	
Missing	0	0	
**Axillary surgery**			0.0025
Sentinel lymph node	468 (57.9)	588 (51.0)	
Axillary dissection	340 (42.1)	565 (49.0)	
Missing	0	0	
**Chemotherapy**			0.0238
No	264 (32.7)	322 (27.9)	
Yes	544 (67.3)	831 (72.1)	
Missing	0	0	
**Radiation therapy**			0.8415
No	59 (7.3)	87 (7.6)	
Yes	748 (92.7)	1065 (92.4)	
Missing	1	1	
**Hormonal Therapy**			<.0001
No	134 (16.6)	199 (17.3)	
Tamoxifen alone	586 (72.8)	712 (61.9)	
Other hormonal therapy	85 (10.6)	239 (20.8)	
Missing	3	3	
**Anti-HER2 therapy**			0.1859
No	688 (85.1)	956 (82.9)	
Yes	120 (14.9)	197 (17.1)	
Missing	0	0	
**Anxiety**			0.9670
Non-case (score 0-7)	263 (33.8)	373 (33.9)	
Doubtful or case (score ≥8)	514 (66.2)	726 (66.1)	
Missing	31	54	

© 2022 Lambertini M et al. *JAMA Network Open*.

**Depression**			0.2246
Non-case (score 0-7)	876 (79.7)	636 (82.0)	
Doubtful or case (score ≥8)	223 (20.3)	140 (18.0)	
Missing	54	32	
**Patient-reported Quality of Life (EORTC QLQ-C30), Mean (SD)****			
Global Health Status	68.7 (17.8)	65.7 (18.6)	0.0012
Physical Function	89.7 (12.0)	85.8 (14.7)	<.0001
Emotional Function	70.1 (24.2)	66.3 (26.0)	0.0033
Cognitive Function	71.9 (26.7)	74.5 (25.5)	0.0519
Social Function	85.6 (20.9)	82.1 (24.4)	0.0018
Role Function	81.2 (23.5)	84.6 (20.9)	0.0023
**Severe Fatigue (EORTC QLQ-C30)****	250 (37.6)	414 (44.0)	0.0103
**Patient-reported Quality of Life (EORTC QLQ-BR23), Mean (SD)****			
Body Image	75.1 (28.2)	70.0 (30.6)	0.0005
Sexual Function	44.3 (23.1)	32.7 (26.5)	<.0001
Sexual Enjoyment	64.0 (26.6)	61.3 (28.7)	0.0989
**Gynecological symptoms (CTCAE v4.0 any grade)****			
Vaginal dryness	318 (39.4)	419 (36.4)	0.1793
Leucorrhea	374 (46.3)	361 (31.3)	<.0001
Hot Flashes	552 (68.3)	883 (76.6)	<.0001
**Consultation with a gynecologist over the previous year (between year-1 and year-2 visits)****			
No	238 (30.4)	410 (36.4)	0.0057
Yes	546 (69.6)	715 (63.6)	
Missing	24	28	

*Chi square test for categorical and t-test for continuous variables. **Collected at year-2 follow-up visit. All the other presented characteristics were collected at breast cancer diagnosis. SD=Standard Deviation; MET=Metabolic Equivalent of Task; HR=Hormone receptor; HER2=Human Epidermal Growth Factor Receptor 2; EORTC QLQ=European Organisation for Research and Treatment of Cancer Quality of Life Questionnaire; CTCAE=Common Terminology Criteria for Adverse Events.

© 2022 Lambertini M et al. *JAMA Network Open*.

eTable 3. Multivariable Logistic Regression Models of Factors Associated With Use of Contraception at Year 1 and Year 2

	Year 1		Year 2	
**Exposure covariates**	**Adjusted* Odds Ratio (95% Confidence Interval)**	**p-value**	**Adjusted* Odds Ratio (95% Confidence Interval)**	**p-value**
**Use of contraception at diagnosis**		<.0001		<.0001
No	Ref.		Ref.	
Yes	4.02 (3.15-5.14)		3.12 (2.36-4.14)	
**Age (continuous)**		<.0001		<.0001
1-year decrease	1.09 (1.07-1.13)		1.11 (1.08-1.15)	
**Marital Status**		0.2804		0.0236
Not in a relationship	Ref.		Ref.	
In a relationship	1.21 (0.85-1.73)		1.61 (1.07-2.44)	
**Highest education level**		0.2314		0.3872
Primary or high school	Ref.		Ref.	
College graduate or higher	1.17 (0.90-1.51)		1.14 (0.85-1.54)	
**Monthly household income**		0.7199		0.8080
<3,000 Euro	Ref.		Ref.	
≥3,000 Euro	0.95 (0.72-1.26)		0.96 (0.69-1.33)	
**Having children**		0.0009		0.8073
No	Ref.		Ref.	
Yes	4.21 (1.80-9.86)		1.10 (0.51-2.39)	
**Tumor stage**		0.2358		0.4763
I	Ref.		Ref.	
II/III	0.85 (0.65-1.11)		0.89 (0.65-1.22)	
**Chemotherapy**		0.1662		0.3498
No	Ref.		Ref.	
Yes	0.80 (0.59-1.09)		0.85 (0.59-1.20)	
**Hormonal Therapy**				
No	1.01 (0.67-1.53)	0.9444	1.32 (0.80-2.16)	0.2772
Tamoxifen alone	1.39 (1.01-1.92)	0.0461	2.16 (1.48-3.15)	<.0001
Other hormonal therapy	Ref.		Ref.	
**Depression**		0.9600		0.8408
Non-case (score 0-7)	Ref.		Ref.	
Doubtful or case (score ≥8)	1.01 (0.74-1.38)		1.04 (0.73-1.48)	
**Patient-reported Quality of Life (EORTC QLQ-C30), continuous 10-point increase****				
Global Health Status	0.98 (0.90-1.07)	0.8254	0.95 (0.85-1.06)	0.3921
Physical Function	1.03 (0.93-1.15)	0.4886	1.08 (0.94-1.24)	0.2613
Emotional Function	0.99 (0.93-1.06)	0.9657	1.03 (0.96-1.11)	0.3917
Cognitive Function	1.01 (0.95-1.07)	0.6231	0.94 (0.88-1.00)	0.0543
Social Function	1.02 (0.96-1.09)	0.1579	1.04 (0.96-1.13)	0.3608

© 2022 Lambertini M et al. *JAMA Network Open*.

**Severe fatigue****		0.7354		0.2351
No	Ref.		Ref.	
Yes	0.92 (0.67-1.25)		0.81 (0.57-1.15)	
**Patient-reported Quality of Life (EORTC QLQ-BR23), continuous 10-point increase****				
Body Image	0.99 (0.95-1.04)	0.9107	1.01 (0.95-1.06)	0.8590
Sexual Function	1.13 (1.07-1.19)	<.0001	1.10 (1.04-1.16)	0.0012
**Leucorrhea****		0.0289		0.0012
No	Ref.		Ref.	
Yes	1.32 (1.03-1.70)		1.59 (1.20-2.10)	
**Hot Flashes****		0.9761		0.1764
No	Ref.		Ref.	
Yes	1.00 (0.75-1.34)		0.80 (0.58-1.10)	
**Consultation with a gynecologist over the previous year****		0.0312		0.0252
No	Ref.		Ref.	
Yes	1.29 (1.02-1.63)		1.39 (1.04-1.86)	

*Adjusted for all factors presented in the table. **These covariates were collected at year-1 for models of use of contraception at year-1 and at year-2 for models of use of contraception at year-2. All the other presented characteristics were collected at breast cancer diagnosis. EORTC QLQ= European Organisation for Research and Treatment of Cancer Quality of Life Questionnaire.

© 2022 Lambertini M et al. *JAMA Network Open*.

eTable 4. Patient Characteristics by Use of Contraception Over Time (n = 1640)

	Use of contraception over time				
**N (%)**	**Never<br>N= 476 (29.0)**	**Persistent use<br>N= 439 (26.8)**	**Discontinuation after diagnosis<br>N= 500 (30.5)**	**Start after diagnosis<br>N= 225 (13.7)**	**p***
**Total**					
**Age at diagnosis, years**					<.0001
Mean (SD)	44.5 (4.8)	41.6 (5.4)	43.7 (5.3)	40.5 (5.0)	
Missing	0	0	0	0	
**Marital Status**					<.0001
Not in a relationship	133 (30.0)	55 (13.1)	95 (19.8)	45 (20.8)	
In a relationship	311 (70.0)	365 (86.9)	384 (80.2)	171 (79.2)	
Missing	32	19	21	9	
**Highest education level**					0.0192
Primary or lower	19 (4.3)	10 (2.4)	18 (3.7)	6 (2.8)	
High school	188 (42.2)	156 (36.8)	215 (44.5)	73 (33.5)	
College graduate or higher	239 (53.6)	258 (60.8)	250 (51.8)	139 (63.8)	
Missing	30	15	17	7	
**Monthly household income**					0.0279
<3,000 Euro	221 (51.6)	169 (41.6)	206 (44.7)	92 (44.4)	
≥3,000 Euro	207 (48.4)	237 (58.4)	255 (55.3)	115 (55.6)	
Missing	48	33	39	18	
**Having children**					<.0001
No	25 (6.3)	2 (0.5)	11 (2.4)	12 (5.8)	
Yes	371 (93.7)	417 (99.5)	448 (97.6)	195 (94.2)	
Missing	80	20	41	18	
**Charlson Comorbidity Index**					0.9985
0	397 (89.2)	378 (89.4)	410 (89.3)	191 (89.7)	
1+	48 (10.8)	45 (10.6)	49 (10.7)	22 (10.3)	
Missing	31	16	41	12	
**Body Mass Index, Kg/m^2**					0.0995
Mean (SD)	24.6 (5.4)	24.1 (4.6)	24.5 (4.7)	23.8 (4.6)	
Missing	0	0	0	0	
**Level of physical activity, MET-h/week**					0.6634
Median (Q1-Q3)	15.0 (1.3–40.0)	16.0 (4.0–44.0)	16.0 (2.7–44.0)	16.0 (0.0–37.5)	
Missing	26	16	16	5	
**Smoking behavior**					0.4896
Current smoker	113 (24.1)	104 (23.9)	129 (26.1)	41 (18.4)	
Former smoker	116 (24.7)	113 (26.0)	117 (23.7)	60 (26.9)	
Never smoker	240 (51.2)	218 (50.1)	248 (50.2)	122 (54.7)	
Missing	7	4	6	2	

© 2022 Lambertini M et al. *JAMA Network Open*.

**Alcohol frequency consumption**					0.8568
Daily	427 (91.2)	392 (91.0)	452 (92.4)	199 (91.3)	
Less-than-daily	41 (8.8)	39 (9.0)	37 (7.6)	19 (8.7)	
Missing	8	8	11	7	
**Tumor stage**					0.0243
I	171 (36.0)	201 (45.9)	203 (40.6)	95 (42.2)	
II/III	304 (64.0)	237 (54.1)	297 (59.4)	130 (57.8)	
Missing	1	1	0	0	
**Tumor subtype**					0.6935
HR+/HER2-	325 (68.9)	319 (73.0)	349 (70.4)	159 (70.7)	
HR+/HER2+	77 (16.3)	64 (14.6)	69 (13.9)	30 (13.3)	
HR-/HER2+	20 (4.2)	12 (2.7)	20 (4.0)	13 (5.8)	
HR-/HER2-	50 (10.6)	42 (9.6)	58 (11.7)	23 (10.2)	
Missing	4	2	4	0	
**Breast surgery**					0.3991
Partial surgery	299 (62.8)	298 (67.9)	318 (63.6)	145 (64.4)	
Mastectomy	177 (37.2)	141 (32.1)	182 (36.4)	80 (35.6)	
Missing	0	0	0	0	
**Axillary surgery**					0.0062
Sentinel lymph node	230 (48.3)	258 (58.8)	251 (50.2)	126 (56.0)	
Axillary dissection	246 (51.7)	181 (41.2)	249 (49.8)	99 (44.0)	
Missing	0	0	0	0	
**Chemotherapy**					0.0744
No	121 (25.4)	146 (33.3)	149 (29.8)	64 (28.4)	
Yes	355 (74.6)	293 (66.7)	351 (70.2)	161 (71.6)	
Missing	0	0	0	0	
**Radiation therapy**					0.8283
No	35 (7.4)	34 (7.7)	41 (8.2)	14 (6.3)	
Yes	440 (92.6)	405 (92.3)	459 (91.8)	210 (93.8)	
Missing	0	0	0	0	
**Hormonal Therapy**					<.0001
No	83 (17.5)	62 (14.2)	101 (20.2)	40 (17.9)	
Tamoxifen alone	293 (61.8)	328 (74.9)	290 (58.0)	157 (70.1)	
Other hormonal therapy	98 (20.7)	48 (11.0)	109 (21.8)	27 (12.1)	
Missing	2	1		1	
**Anti-HER2 therapy**					0.3020
No	386 (81.1)	374 (85.2)	374 (85.2)	186 (82.7)	
Yes	90 (18.9)	65 (14.8)	65 (14.8)	39 (17.3)	
Missing	0	0	0	0	
**Anxiety**					0.5943
Non-case (score 0-7)	148 (33.1)	135 (31.8)	174 (35.9)	76 (35.0)	
Doubtful or case (score ≥8)	299 (66.9)	289 (68.2)	311 (64.1)	141 (65.0)	

© 2022 Lambertini M et al. *JAMA Network Open*.

Missing	29	15	15	8	
**Depression**					0.2374
Non-case (score 0-7)	351 (78.5)	342 (80.9)	404 (83.3)	181 (83.4)	
Doubtful or case (score ≥8)	96 (21.5)	81 (19.1)	81 (16.7)	36 (16.6)	
Missing	29	16	15	8	
**Patient-reported Quality of Life (EORTC QLQ-C30), Mean (SD)**					
Global Health Status	67.5 (19.9)	68.1 (17.7)	68.5 (18.9)	66.3 (17.0)	0.2703
Physical Function	92.6 (11.9)	96.1 (8.1)	93.2 (12.3)	94.5 (9.1)	<.0001
Emotional Function	60.1 (24.9)	59.5 (24.0)	62.9 (24.1)	57.9 (24.7)	0.0492
Cognitive Function	78.6 (4.4)	80.2 (22.3)	79.7 (22.6)	78.3 (23.5)	0.6850
Social Function	87.5 (21.3)	90.3 (17.6)	89.5 (20.0)	88.2 (19.1)	0.1599
Role Function	83.7 (23.5)	86.9 (20.5)	84.4 (24.3)	83.5 (24.0)	0.1508
Severe Fatigue, N (%)	158 (34.9)	124 (29.4)	152 (31.6)	85 (38.6)	0.0782
**Patient-reported Quality of Life (EORTC QLQ-BR23), Mean (SD)**					
Body Image	86.6 (20.8)	87.4 (19.8)	87.5 (20.1)	85.4 (22.5)	0.5841
Sexual Function	28.2 (6.6)	40.1 (24.8)	34.7 (26.2)	38.1 (26.4)	<.0001
Sexual Enjoyment	65.1 (9.7)	69.9 (24.8)	67.1 (26.9)	66.7 (25.2)	0.2979
**Gynecological symptoms (CTCAE v4.0 any grade)**					
Vaginal dryness	52 (11.3)	40 (9.3)	46 (9.6)	24 (11.1)	0.7017
Leucorrhea	165 (36.0)	176 (41.3)	152 (31.9)	82 (37.8)	0.0329
Hot Flashes	94 (20.5)	43 (9.9)	80 (16.7)	33 (15.1)	0.0002

*Chi square test for categorical and t-test for continuous variables. All the presented characteristics were collected at breast cancer diagnosis. EORTC QLQ= European Organisation for Research and Treatment of Cancer Quality of Life Questionnaire.

© 2022 Lambertini M et al. *JAMA Network Open*.

eTable 5. Multivariable Multinomial Logistic Regression Models of Use of Contraception Over Time (n = 1640)

	**Use of contraception over time (vs. Never [Ref.], N=476 [29.0%])**					
**Exposure covariates**	**Always N=439 (26.8)**		**Discontinuation after diagnosis N=500 (30.5)**		**Start after diagnosis N=225 (13.7)**	
	**Adjusted* Odds Ratio (95% Confidence Interval)**	**p-value**	**Adjusted* Odds Ratio (95% Confidence Interval)**	**p-value**	**Adjusted* Odds Ratio (95% Confidence Interval)**	**p-value**
**Age (continuous)**		<.0001		0.0050		<.0001
1-year decrease	1.14 (1.09-1.18)		1.06 (1.02-1.09)		1.18 (1.13-1.23)	
**Marital Status**						
Not in a relationship	Ref.	0.0002	Ref.	0.0871	Ref.	0.7611
In a relationship	2.58 (1.57-4.23)		1.45 (0.947-2.23)		1.09 (0.64-1.85)	
**Highest education level**						
Primary or high school	Ref.	0.4673	Ref.	0.7681	Ref.	0.4610
College graduate or higher	1.14 (0.80-1.63)		0.95 (0.68-1.33)		1.17 (0.77-1.79)	
**Monthly household income**						
<3,000 Euro	Ref.	0.7306	Ref.	0.5869	Ref.	0.6343
≥3,000 Euro	0.93 (0.63-1.38)		1.11 (0.76-1.61)		1.12 (0.70-1.78)	
**Having children**						
No	Ref.	0.0003	Ref.	0.0114	Ref.	0.0766
Yes	16.46 (3.65-74.26)		2.75 (1.26-6.02)		2.28 (0.91-5.70)	
**Tumor stage**						
I	Ref.	0.0083	Ref.	0.2913	Ref.	0.0845
II/III	0.64 (0.46-0.89)		0.84 (0.61-1.16)		0.71 (0.48-1.05)	
**Hormonal Therapy**						
No	1.08 (0.60-1.94)	0.8035	1.06 (0.65-1.73)	0.8149	1.38 (0.68-2.80)	0.3645
Tamoxifen alone	2.07 (1.32-3.26)	0.0016	0.98 (0.67-1.43)	0.9265	2.23 (1.26-3.93)	0.0059
Other hormonal therapy	Ref.		Ref.		Ref.	
**Patient-reported Quality of Life, continuous 10-point increase**						
Physical Function	1.36 (1.14-1.62)	0.0008	1.03 (0.90-1.18)	0.6714	1.15 (0.96-1.39)	0.1267
Emotional Function	0.95 (0.88-1.01)	0.1248	1.04 (0.97-1.11)	0.2728	0.97 (0.89-1.05)	0.4362
**Patient-reported Quality of Life (EORTC QLQ-BR23), continuous 10-point increase**						
Sexual Function	1.12 (1.05-1.19)	0.0007	1.07 (1.01-1.14)	0.0204	1.07 (0.99-1.16)	0.0633
**Leukorrhea**						
No	Ref.	0.2135	Ref.	0.1649	Ref.	0.8490
Yes	1.24 (0.88-1.73)		0.79 (0.57-1.10)		0.96 (0.65-1.43)	
**Hot Flashes**		0.0299		0.9371		0.4421
No	Ref.		Ref.		Ref.	

© 2022 Lambertini M et al. *JAMA Network Open*.

Yes	0.59 (0.36-0.95)		1.02 (0.68-1.51)		1.23 (0.73-2.08)	

*Adjusted for all factors presented in the table. All the presented characteristics were collected at breast cancer diagnosis. EORTC QLQ= European Organisation for Research and Treatment of Cancer Quality of Life Questionnaire.

© 2022 Lambertini M et al. *JAMA Network Open*.

eTable 6. Sensitivity Analysis Among Patients Younger Than 45 Years at Diagnosis: Multivariable Logistic Regression Models of Factors Associated With Use of Contraception at Year 1 and Year 2

	Year 1 (n=1,507)		Year 2 (n=1,507)	
**Exposure covariates**	**Adjusted* Odds Ratio (95% Confidence Interval)**	**p-value**	**Adjusted* Odds Ratio (95% Confidence Interval)**	**p-value**
**Use of contraception at diagnosis**		<.0001		<.0001
No	Ref.		Ref.	
Yes	4.16 (2.97-5.82)		2.74 (1.86-4.03)	
**Age (continuous)**		.0004		<.0001
1-year decrease	1.08 (1.04-1.13)		1.14 (1.08-1.20)	
**Marital Status**		0.4287		0.1397
Not in a relationship	Ref.		Ref.	
In a relationship	1.22 (0.75-1.97)		1.55 (0.87-2.75)	
**Highest education level**		0.0214		0.1551
Primary or high school	Ref.		Ref.	
College graduate or higher	1.54 (1.07-2.21)		1.36 (0.89-2.08)	
**Monthly household income**		0.8119		0.6171
<3,000 Euro	Ref.		Ref.	
≥3,000 Euro	0.95 (0.65-1.41)		1.12 (0.72-1.74)	
**Having children**		0.0011		0.3434
No	Ref.		Ref.	
Yes	5.23 (1.94-14.14)		1.61 (0.60-4.34)	
**Tumor stage**		0.5041		0.6894
I	Ref.		Ref.	
II/III	0.88 (0.61-1.27)		0.92 (0.60-1.41)	
**Chemotherapy**		0.3697		0.2338
No	Ref.		Ref.	
Yes	0.81 (0.51-1.29)		0.73 (0.43-1.23)	
**Hormonal Therapy**				
No	0.82 (0.46-1.44)	0.4869	1.53 (0.76-3.06)	0.2300
Tamoxifen alone	1.22 (0.76-1.97)	0.4049	2.66 (1.52-4.66)	.0006
Other hormonal therapy	Ref.		Ref.	
**Depression**		0.7187		0.5206
Non-case (score 0-7)	Ref.		Ref.	
Doubtful or case (score ≥8)	1.08 (0.71-1.63)		0.85 (0.53-1.38)	
**Patient-reported Quality of Life (EORTC QLQ-C30), continuous 10-point increase****				
Global Health Status	0.97 (0.86-1.09)	0.5998	0.93 (0.79-1.09)	0.3623
Physical Function	1.00 (0.86-1.16)	0.9694	1.16 (0.96-1.42)	0.1282
Emotional Function	0.97 (0.89-1.06)	0.5536	1.08 (0.97-1.21)	0.1526
Cognitive Function	1.02 (0.95-1.10)	0.6118	0.91 (0.83-1.00)	0.0526

© 2022 Lambertini M et al. *JAMA Network Open*.

Social Function	1.03 (0.94-1.12)	0.5525	0.96 (0.86-1.08)	0.5316
**Severe fatigue****		0.5759		0.2728
No	Ref.		Ref.	
Yes	0.89 (0.58-1.35)		0.77 (0.48-1.24)	
**Patient-reported Quality of Life (EORTC QLQ-BR23), continuous 10-point increase****				
Body Image	1.03 (0.97-1.09)	0.4028	1.00 (0.93-1.08)	0.9218
Sexual Function	1.15 (1.07-1.23)	<.0001	1.07 (0.98-1.16)	0.1156
**Leucorrhea****		0.1167		0.0219
No	Ref.		Ref.	
Yes	1.32 (0.93-1.86)		1.56 (1.07-2.29)	
**Hot Flashes****		0.6331		0.2807
No	Ref.		Ref.	
Yes	1.10 (0.74-1.65)		0.79 (0.51-1.22)	
**Consultation with a gynecologist over the previous year****		0.2901		0.2752
No	Ref.		Ref.	
Yes	1.19 (0.86-1.65)		1.25 (0.84-1.88)	

*Adjusted for all factors presented in the table. **These covariates were collected at year-1 for models of use of contraception at year-1 and at year-2 for models of use of contraception at year-2. All the other presented characteristics were collected at breast cancer diagnosis. EORTC QLQ= European Organisation for Research and Treatment of Cancer Quality of Life Questionnaire.

© 2022 Lambertini M et al. *JAMA Network Open*.

eTable 7. Sensitivity Analysis Among Patients Who Reported They Were Sexually Active (Defined as Sexual Function Score >0 Points): Multivariable Logistic Regression Models of Factors Associated With Use of Contraception at Year 1 and Year 2

	Year 1 (n=1,877)		Year 2 (n=1,574)	
**Exposure covariates**	**Adjusted* Odds Ratio (95% Confidence Interval)**	**p-value**	**Adjusted* Odds Ratio (95% Confidence Interval)**	**p-value**
**Use of contraception at diagnosis**		<.0001		<.0001
No	Ref.		Ref.	
Yes	3.90 (2.30-5.08)		3.10 (2.29-4.20)	
**Age (continuous)**		<.0001		<.0001
1-year decrease	1.09 (1.06-1.12)		1.11 (1.08-1.15)	
**Marital Status**		0.8848		0.3426
Not in a relationship	Ref.		Ref.	
In a relationship	0.97 (0.65-1.45)		1.25 (1.79-1.99)	
**Highest education level**		0.0893		0.4475
Primary or high school	Ref.		Ref.	
College graduate or higher	1.27 (0.96-1.68)		1.13 (0.82-1.56)	
**Monthly household income**		0.3840		0.6456
<3,000 Euro	Ref.		Ref.	
≥3,000 Euro	0.87 (0.65-1.18)		0.92 (0.65-1.31)	
**Having children**		0.0016		0.6915
No	Ref.		Ref.	
Yes	4.34 (1.75-10.80)		1.18 (0.51-2.73)	
**Tumor stage**		0.0795		0.3275
I	Ref.		Ref.	
II/III	0.77 (0.58-1.03)		0.85 (0.61-1.18)	
**Chemotherapy**		0.3015		0.2889
No	Ref.		Ref.	
Yes	0.84 (0.60-1.17)		0.81 (0.56-1.19)	
**Hormonal Therapy**				
No	1.01 (0.65-1.58)	0.9564	1.16 (0.68-1.98)	0.5777
Tamoxifen alone	1.30 (0.91-1.84)	0.1464	1.98 (1.32-2.96)	.0010
Other hormonal therapy	Ref.		Ref.	
**Depression**		0.5438		0.3426
Non-case (score 0-7)	Ref.		Ref.	
Doubtful or case (score ≥8)	1.11 (0.79-1.56)		1.21 (0.82-1.79)	
**Patient-reported Quality of Life (EORTC QLQ-C30), continuous 10-point increase****				
Global Health Status	1.00 (0.91-1.10)	0.9107	0.97 (0.86-1.09)	0.5862
Physical Function	1.04 (0.93-1.17)	0.4969	1.09 (0.94-1.27)	0.2592
Emotional Function	1.00 (0.94-1.08)	0.9136	1.04 (0.97-1.13)	0.2840
Cognitive Function	1.00 (0.94-1.07)	0.9712	0.92 (0.86-0.99)	0.0292

© 2022 Lambertini M et al. *JAMA Network Open*.

Social Function	1.04 (0.97-1.11)	0.2938	1.06 (0.97-1.17)	0.1827
**Severe fatigue****		0.3892		0.2246
No	Ref.		Ref.	
Yes	0.86 (0.62-1.21)		0.79 (0.54-1.15)	
**Patient-reported Quality of Life (EORTC QLQ-BR23), continuous 10-point increase****				
Body Image	0.98 (0.94-1.03)	0.5126	0.99 (0.94-1.06)	0.8676
**Leucorrhea****		0.0281		0.0006
No	Ref.		Ref.	
Yes	1.35 (1.03-1.78)		1.70 (1.26-2.30)	
**Hot Flashes****		0.9622		0.2929
No	Ref.		Ref.	
Yes	1.01 (0.74-1.37)		0.83 (0.59-1.17)	
**Consultation with a gynecologist over the previous year****		0.0297		0.0247
No	Ref.		Ref.	
Yes	1.32 (1.03-1.70)		1.43 (1.05-1.95)	

*Adjusted for all factors presented in the table. **These covariates were collected at year-1 for models of use of contraception at year-1 and at year-2 for models of use of contraception at year-2. All the other presented characteristics were collected at breast cancer diagnosis. EORTC QLQ= European Organisation for Research and Treatment of Cancer Quality of Life Questionnaire.

© 2022 Lambertini M et al. *JAMA Network Open*.
